# Molecular imaging in masseter muscle observed by muscle function magnetic resonance imaging and ^31^P‐magnetic resonance spectroscopy in patients with a jaw deformity

**DOI:** 10.1002/cre2.494

**Published:** 2021-10-08

**Authors:** Masahiro Arakawa, Toru Kitahara, Daisuke Inadomi, Masahiro Iikubo, Hiroto Hyakutake, Kenji Yuasa, Ichiro Takahashi

**Affiliations:** ^1^ Section of Orthodontics and Dentofacial Orthopedics, Graduate School of Dentistry Kyushu University Fukuoka Japan; ^2^ Section of Orthodontics and Dentofacial Orthopedics, Faculty of Dental Science Kyushu University Fukuoka Japan; ^3^ Radiology Center, Fukuoka Dental College Hospital Fukuoka Japan; ^4^ Department of Oral Diagnosis Tohoku University Graduate School of Dentistry Sendai Japan; ^5^ Graduate School of Information Sciences Hiroshima City University Hiroshima Japan; ^6^ Section of Image Diagnosis, Department of Diagnostics and General Care Fukuoka Dental College Fukuoka Japan

**Keywords:** functional MRI, magnetic resonance spectroscopy, masseter muscle, molecular imaging, muscle fatigue, prognathism

## Abstract

**Background:**

Skeletal mandibular protrusion would influence to the muscle fatigue of the masticatory muscles. Establishing a diagnostic procedures combining physiological and biochemical information is necessary for quantitative evaluation of masticatory muscle fatigue.

**Objective:**

The transverse relaxation time (T2 time) of muscle functional magnetic resonance imaging (mfMRI), and ^31^P‐magnetic resonance spectroscopy (MRS) were used to investigate the reliability as parameters for measuring the masseter muscle in patients with skeletal mandibular prognathism.

**Method:**

The subjects were 19 patients diagnosed as skeletal mandibular protrusions and 19 healthy subjects as a control group. Transverse relaxation time (T2 value) determined by mfMRI along with creatine phosphate (PCr) and inorganic phosphorus (Pi) determined by ^31^P‐MRS before, during, and after clenching were used for molecular imaging of muscle fatigue.

**Results:**

The average T2 value of the patient group was significantly higher than that of the healthy control group at rest. Furthermore, the average T2 value transiently increased in both groups during experimental clenching. The PCr and Pi showed a tendency toward a transient decrease and increases, respectively. The pH in the masseter muscle showed a transient decrease in both groups prior to and following experimental clenching. The pH in the masseter muscle of the patient group was significantly lower than that in the healthy control group at rest and recovery.

**Conclusion:**

We showed mfMRI and ^31^P‐MRS are useful for evaluating masseter fatigue during clenching, and the masseter muscle in the prognathic patients showed more severe fatigue than the healthy controls.

## 1 INTRODUCTION

Muscle fatigue and pain in the masseter and temporal muscles are generally of great concern with regard to the quality of life. Masticatory muscle fatigue and pain are recognized as causing lockjaw, and masticatory dysfunction, and pain, greatly affecting the medical endpoint of “improving the quality of life” (Bock et al., 2009; Cunningham et al., 2000; Eslamipour et al., 2017; Sun et al., 2018). Masticatory muscle fatigue is frequently reported among cases of masticatory dysfunctions in patients with jaw deformities (Rowlerson et al., 2005; Sciote et al., 2012). Many patients who undergo orthognathic surgery are diagnosed having masticatory muscle fatigue based on the level of self‐assessment and their own complaints of pain, without an effective objective or quantitative evaluation, highlighting the urgent need to establish a diagnostic method that combines physiological and biochemical information.

Molecular imaging by magnetic resonance imaging (MRI) enables the visualization of the intra‐individual molecular movements inside a live body without damaging the individual. Thus far, for skeletal muscles with large cross‐sectional areas, such as the quadriceps muscles, molecular imaging has been carried out using muscle functional MRI (mfMRI) (Akima et al., 2004; Cagnie et al., 2011; Kinugasa & Akima, 2005; Segal, 2007) and phosphorus‐31 nuclear magnetic resonance spectroscopy (^31^P‐MRS) (Hoff et al., 2013; Stutzig et al., 2016; van Oorschot et al., 2013), which are both noninvasive examinations. These techniques allow physiological information and biochemical information of the muscles to be obtained simultaneously enabling a comparison of their findings.

MfMRI is a noninvasive technique for investigating muscle activation and is frequently used to determine spatial patterns of muscular involvement in exercising humans. Tissue contrast in magnetic resonance images is dependent on proton density and the physical parameters spin–lattice and spin–spin relaxation times (T2). Increases in T2 are known to occur during exercise in concert with increases in metabolite accumulation and fluids concert with increases in metabolite accumulation and fluid shifts that increase the muscle extracellular fluid volume (Hargreaves et al., 1998; Meyer & Prior, 2000). Several studies have demonstrated the close relationship between the masticatory muscular fiber activation and the increase in the T2 relaxation time (Chikui et al., 2010; Nikkuni et al., 2013; Shiraishi et al., 2011). The technique is based on the acquisition of T2‐weighted MR images and the subsequent quantitation of spin–spin transverse relaxation times (T2) before and after muscular loading. While electromyography is a widely accepted method for studying local muscle activity, mfMRI is less invasive and allows for the determination of the whole muscle response, which is often difficult to access in human subjects.

There is an increasing need for advanced noninvasive technologies to quantify masticatory muscle fatigue in order to assess the functional and biomechanical properties in vivo. The T2 relaxation times obtained on mfMRI of the muscles reflect micro molecular environmental changes in the muscles; this parameter reflects environmental water binding and is prolonged by increased amounts of less bounded water (Fisher, 1990; Fleckenstein et al., 1988).

^31^P‐MRS is a powerful tool for evaluating the energetic metabolism of phosphoric acid in the skeletal muscles. Magnetic resonance spectroscopy (MRS) gives a unique view of tissue biochemistry in site by measuring the concentrations and/or turnover rates of metabolites, and has been widely applied to skeletal muscle. In the MR 'spectrum' a range of different metabolites is separately detected, because the different magnetic environments of nuclei in different molecules or molecular sites shift their resonance frequencies (Kemp, 1994; Prompers et al., 2006). Applying ^31^P‐chemical shift imaging enables the detection and quantitation of these metabolic changes locally, including inorganic phosphate (Pi), creatine phosphate (PCr), adenosine triphosphate (ATP), and intra‐cellular pH (Taylor et al., 1983). The chemical shifts in each peak of the spectrum indicate the molecular type, whereas the peak dimensions reflect the abundance thereof. ^31^P‐MRS enables the noninvasive and repeated measurement of the concentrations of high‐energy phosphates, allowing the evaluation of the masticatory muscle function through such measurements (Al‐Farra et al., 2001; Okada et al., 2005).

The skeletal muscle oxidative capacity may be measured in vivo using ^31^P‐MRS by monitoring the recovery of phosphocreatine (PCr) following prior depletion by a stimulus, such as exercise. As described previously, the intracellular pH can be determined from the chemical shift difference between the PCr and Pi signals (Gadian & Robinson, 1979; Hoult et al., 1974; Lanza et al., 2011).

Dynamic MR spectroscopy is one of the most insightful techniques for investigating the metabolism in living tissues. An increase in the spectral and temporal resolution on ^31^P‐MRS indicates a new potential to quantitative muscle fatigue of each of these ATP‐generating activities in vivo. The intracellular pH can be determined from the difference in the chemical shift between the PCr and Pi signals.

Skeletal muscle exercises cause an increase in hydrostatic pressure on the capillary wall due to increased blood flow, along with an increase in extravascular osmotic pressure and extravascular movement of plasma due to an increase in metabolites such as lactic acid, resulting in increased muscle water content. Furthermore, the intracellular pH of muscle cells is maintained at approximately pH 7.1 at rest (Aickin & Thomas, 1977). However, because H^+^ is produced by ATP hydrolysis and glycolysis in the process of muscle contraction, the intracellular pH of muscle cells temporarily decreases. In this study, the T2 value of whole muscles is measured using mfMRI, which has high‐spatial resolution for these phenomena, while the decrease in pH is detected using ^31^P‐MRS, which has high‐temporal resolution. Integrating these two methods makes it possible to visualize the movement of molecules throughout the living body of an individual, as molecular imaging for muscle fatigue, without damaging the individual.

The objective of this study was to assess the validity of this new diagnostic imaging method using molecular imaging for the diagnosis of acute and transient muscle fatigue in the masticatory muscles, which have a relatively small cross‐sectional area, and to establish a method for diagnosing masticatory muscle fatigue. We attempted to establish a method for measuring masseter fatigue using the transverse relaxation time (T2 value) as determined by mfMRI, along with the creatine phosphate (PCr) and inorganic phosphorus (Pi) values as determined by ^31^P‐MRS. We established the following hypotheses: (1) molecular imaging of the masseter muscle with its relatively small cross‐sectional area is a valid approach for diagnosis of masticatory muscle fatigue; (2) regarding mfMRI, the T2 value of the masseter muscle transiently increases during clenching, and the T2 value of the patient group is longer than that in the healthy control group; and (3) when high‐energy phosphate compounds in the masseter muscle are assessed using ^31^P‐MRS prior to and following clenching, the PCr intensity transiently decreases, while the Pi intensity transiently increases. (4) The pH of the masseter muscle showed a transient decreasing trend in both groups prior to and following clenching.

## 2 MATERIALS AND METHODS

### 2.1 Participants

This study was approved by the Ethics Committee of Kyusyu University (reference number: 27066). The inclusion criteria for the patient group were as follows: ≥20 years of age and written agreement for orthognathic surgical treatment obtained from the patient. The maxillofacial morphology was classified based on the ANB angle and Wits appraisal into skeletal Class III (ANB angle of ≤0° or Wits appraisal ≤−5 mm). The exclusion criteria were as follows: presence of a pacemaker or medical metal devices in the heart, temporomandibular disorder, tattoos, claustrophobia, pregnancy, intellectual impairment or psychogenic disorder or deemed inappropriate for inclusion in a medical study. We publicly invited participation from patient ≥20 years of age and obtained informed consent from all subjects.

The volunteers were limited to those ≥20 years of age and able to provide their informed consent to participate. The exclusion criteria for healthy volunteers were presence of anterior cross‐bite.

As there were no marked gender differences in the preliminarily collected data obtained from five males and seven females, the male and female data were integrated. Subsequently, we confirmed that data from a total of 19 people, including males and females, are required, using a variance computed based on the data of the abovementioned 12 people, with the power of test as 90% or greater when the difference in the population mean of the PCr signal strength is 0.75 or greater. A total of 19 patients (12 women and 7 men) who ranged from 20 to 56 years of age and 19 healthy volunteers (6 women and 13 men) who ranged from 22 to 37 years of age participated in this study.

### 2.2 Experimental load for masseter muscle

Maximum voluntary contraction force (MVC) of each subject was monitored using a pressure measuring system (I‐SCAN TM, Nitta, Osaka, Japan). We postulated and retained 30% MVC force during the MR examination prior to the T2 value and MRS measurements. First, the participants were instructed to clench with the 30% of the maximum voluntary contraction (MVC) level and to keep the same level during a clenching period (5 min) with a tactile system for measurement of pressure distribution (I‐SCAN™, Nitta, Osaka, Japan). For loading the masseter muscles, a urethane block (20 × 9 × 24 mm in size, FL‐400; Hanshin Technical Laboratory Ltd., Hyogo, Japan) was placed on the molars, and 30% MVC was continued for 5 min. Dynamic changes in the T2 value, and PCr and Pi signal intensity in the masseter muscle were then measured on MR and ^31^P spectra during rest, clenching and the subsequent recovery period for 20 min.

### 2.3 mfMRI measurements

MR image acquisition was performed at Fukuoka Dental Collage. All the examinations were performed on a 1.5 T scanner (Intera Achieva nova dual; Philips Medical Systems, Eindhoven, the Netherlands) that used a sensitivity‐encoding SENSE‐Flex‐M coil. The muscle functional acquisitions were contiguous 4‐mm axial scans collected from the cricoid to the hard palate utilizing a spin‐echo sequence with a repetition time (TR) of 2000 ms and dual echo times (TE) of 10, 20, 30, 40, 50, 60, 70, and 80 ms. We obtained the T2 maps using the fast spin‐echo (FSE) sequence before, during, immediately after, and 20 min after clenching.

**Figure 1:**
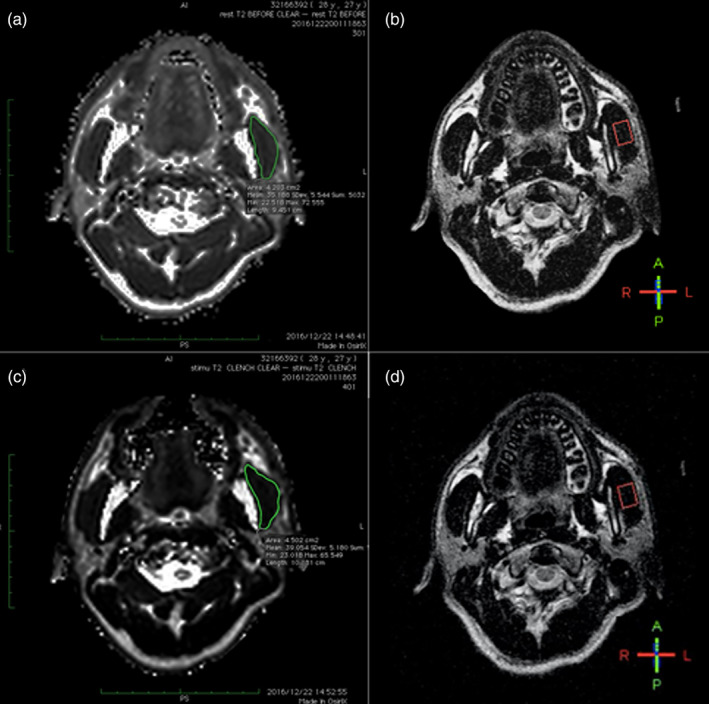
(a, c) Representative axial plane T2‐weighted MR images obtained at the midbelly of the masseter muscle at rest (a) and after clenching (c). Measurement of the T2 value and cross‐sectional area of the masseter muscle. The region of interest (ROI) on a defined axial slice (green line) was measured. (b, d) the position of the ROI on ^31^P‐MRS for the masseter muscle was set so as to obtain as large a pixel size as possible (red line)

T2‐weighted MR images were obtained before and immediately after each motor task (at rest and after clenching) to measure any changes in the T2 values of the masseter muscles induced by clenching (Figure 1a,c). An average T2 of the anatomic region of interest (ROI) was determined by digitizing the region of the enhanced masseter muscle, which provided a computer‐generated average of T2. Digitizing was performed by one investigator (M.A.). Muscle segmentation was carried out using Osirix (v.3.9.4 32‐bit), a software program for digital imaging and communication in medicine.

### 2.4 ^31^P‐MRS and pH measurements

The spectra were obtained using a 10‐cm‐diameter surface coil, placed directly above the masseter muscle belly. ^31^P‐MRS was performed to obtain localizing signals, and then the position of the region of interest (ROI) was chosen. In all subjects, the ROI was located in the center of the masseter muscle, taking care not to affect the values by including different parts of the masseter muscle in all subjects (Figure 1b,d). We fixed the patients head and we controlled the timing of the clenching using a microphone. Slices were selected by a slice‐selective gradient in the long‐axis direction for the masseter muscle and in the sagittal axis direction for the medial pterygoid, and two‐dimensional chemical shift imaging (2D‐CSI) was performed with each of 16 phases encoding in two dimensions. The slice thickness was 30 mm, and the focus of view was 320 mm. The voxel dimensions after *k*‐space 0 filling were 1 × 2 × 3 cm^3^ (1 × 1.5 × 2 cm^3^). For all metabolites aside from PCr, a Lorentzian line shape was assumed. For the PCr peak, we used a combination of Lorentzian and Gaussian line shapes.

The intracellular pH can be determined from the difference in the chemical shift between the PCr and Pi signals. The intramuscular pH was calculated based on the chemical shift (*σ*) of Pi relative to PCr in parts per million (Gadian & Robinson, 1979; Hoult et al., 1974; Lanza et al., 2011):

$$\mathrm{pH}:6.75+\log\;\left(\sigma -3.27\right)/\left(5.69-\sigma \right).$$

### 2.5 Statistical analyses

In order to statistically explain the transient shifts in the measurements of the T2 values, PCr and Pi, Scheffe's multiple comparison was used to measure the values at a total of six time points (rest → clench → rest → rest → rest → rest), and then the hypotheses were verified. The mean data were evaluated by Scheffe's *F*‐test with the level of significance set at *p* < 0.05. For intergroup comparisons, Student's *t*‐test was used to assess the differences between the healthy subjects and the patients. All procedures were conducted using the SPSS software program, ver. 23 (SPSS Inc., Chicago, IL, USA).

## 3 RESULTS

In order to assess reproducibility, we again performed mfMRI and ^31^P‐MRS for five healthy individuals. The data of each individual was within the mean ± 2SD of the data of the 19 subjects. Regarding the Cephalo analysis, tracing was conducted again. The results were within the average ± 2SD of the data of the 19 subjects. Characteristics of the participants are shown in Table 1. The skeletal class III patients (patient group) and healthy controls (healthy control group) had similar ages. An analysis of the correlation of age with T2 value, PCr, and Pi indicated no strong correlation between age and the other items. The ratio of men to women was 7:12 in the patient group and 13:6 in the healthy control group. When a *t*‐test was performed between male and female, no significant difference was found. In the skeletal class III patients, the average ANB angle was −2.2° ± 3.2°, and the average Wits appraisal was −11.2 ± 5.8 mm. A two‐way analysis of variance (ANOVA) was performed on the T2 value, PCr, and Pi signal intensity separately to evaluate the effect of timing after clench release. No significant difference was found at four different endpoints (rest → rest → rest → rest), so we decided to select the data at 20 min.

**Table 1:** Characteristics of the participants

	Healthy controls		Patients	
	Mean	SD	Mean	SD
Age (years)	27.8	4.4	29.4	9.2
Gender (number of subject)				
Female	6		12	
Male	13		7	
ANB angle (°)	―	―	−2.2	3.2
Wits appraisal (mm)	―	―	−11.2	5.8

Abbreviation: SD, standard deviation.

**Figure 2:**
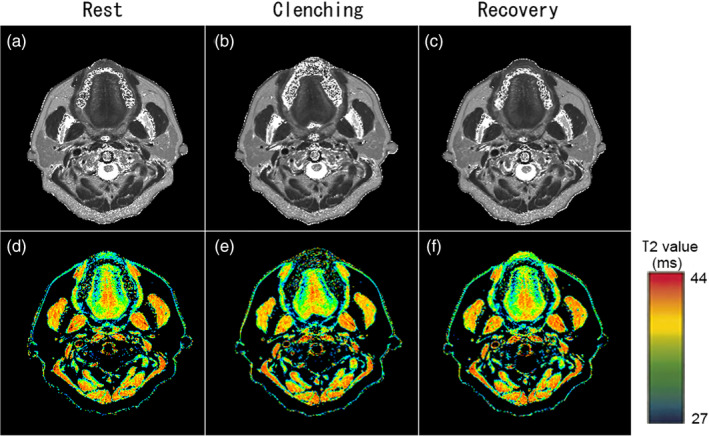
Representative axial T2‐weighted magnetic resonance images (a–c) and T2 mapping images (d–f)

Figure 1a,c shows the T2‐weighted MR images obtained at the midbelly of the masseter muscle at rest and following clenching. The mean values of T2 in this individual were 35.2 and 39.1 ms, at rest and clenching, respectively. The cross‐sectional area of the masseter muscle was 623.6 mm^2^. Figure 2 shows examples of axial T2‐weighted magnetic resonance images (a–c) and colored‐reconstructed T2 mapping images (d–f) at rest, during clenching and in the subsequent recovery period.

**Figure 3:**
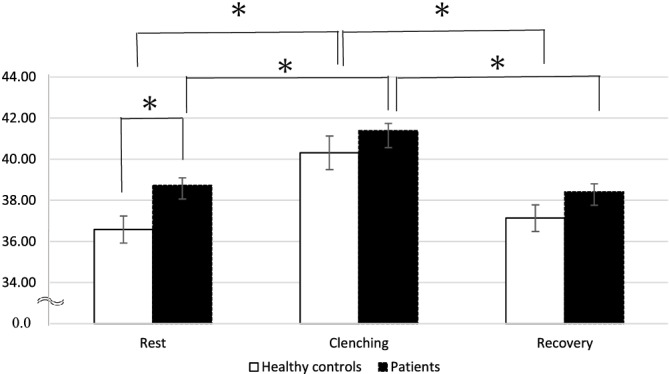
The average T2 value of the patient group and that of the healthy group Scheffe's *F*‐test with the level of significance set at *p* < 0.05

The mean values and SEs (standard error) of the masseter muscle T2 signal profile are shown in Table 2. The mean T2 values at rest in the masseter muscle of the healthy control group and patient group were 36.58 and 38.72 ms, respectively. The mean T2 value in the patient group was significantly higher than that in the healthy control group at rest (*p* < 0.05) (Figure 3). The average T2 value of the masseter muscles increased after clenching on mfMRI. The average T2 value significantly increased in both groups during experimental clenching (*p* < 0.05). As shown in Figure 3, significant changes in the mean T2 value were observed during the recovery period (*p* < 0.05).

**Table 2:** T2 signal profile (m/s) and PCr, Pi signal intensity, and pH in the masseter muscle

	Rest			Clenching			Recovery			Multiple comparisons
	Mean		SE	Mean		SE	Mean		SE	
T2										
Healthy controls	36.58	±	0.66	40.31	±	0.82	37.14	±	0.65	(Rest/clenching, clenching/recovery)*
Patients	38.72	±	0.37	41.39	±	0.36	38.41	±	0.40	(Rest/clenching, clenching/recovery)*
PCr										
Healthy controls	1.43	±	0.14	0.60	±	0.08	1.23	±	0.11	(Rest/clenching, clenching/recovery)*
Patients	1.23	±	0.13	0.54	±	0.07	1.06	±	0.10	(Rest/clenching, clenching/recovery)*
Pi										
Healthy controls	0.16	±	0.02	0.68	±	0.08	0.20	±	0.02	(Rest/clenching, clenching/recovery)*
Patients	0.19	±	0.04	0.78	±	0.15	0.22	±	0.03	(Rest/clenching, clenching/recovery)*
pH										
Healthy controls	7.09	±	0.01	6.95	±	0.01	7.05	±	0.01	(Rest/clenching, clenching/recovery)*
Patients	7.02	±	0.02	6.93	±	0.02	7.00	±	0.02	(Rest/clenching, clenching/recovery)*

Abbreviation: SE, standard error.

* *p* < 0.05.

**Figure 4:**
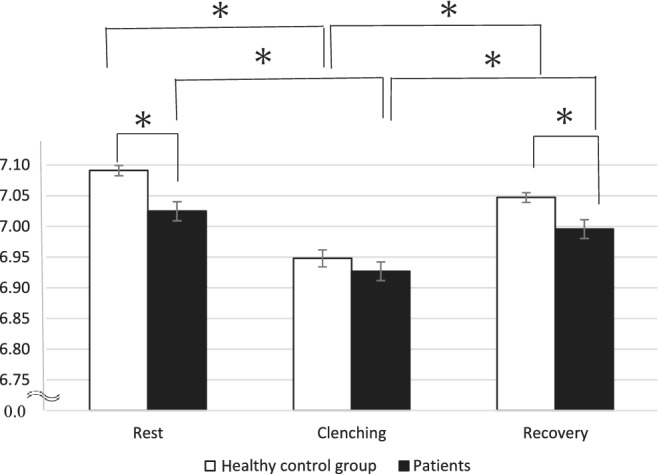
The pH of the patient group and healthy control group in the masseter muscle (*p* < 0.05)

Table 2 shows the mean values and SEs of the PCr and Pi signal intensity as well as the pH in the masseter muscle. The signal of PCr showed a transient decreasing trend in both groups prior to and following experimental clenching (*p* < 0.05). Furthermore, both groups showed a transient increase tendency for Pi (*p* < 0.05). Figure 4 shows the changes in the pH levels during the experimental load in the masseter muscle of the patient and healthy control groups. A significantly lower pH during rest and recovery was observed in the patient group, while this difference decreased during clenching (*p* < 0.05). The pH level in both the patient and healthy control groups showed a transient decrease prior to and following experimental clenching (*p* < 0.05).

The cross‐sectional area of the masseter muscles in the patient group was an average of 320.5 ± 61.6 mm^2^ while that in the healthy control group was an average of 427.6 ± 142 mm^2^. The cross‐sectional area of the patient group was significantly lower than that of the control group (*p* < 0.05).

## 4 DISCUSSION

In the present study, ^31^P‐MRS and T2‐weighted MR images were found to be useful for evaluating the fatigue of masseter muscles. The average T2 value significantly and transiently increased in both healthy controls and patients with jaw deformities during experimental clenching. This increase in T2 values is driven primarily by water shifts resulting from transient changes in tissue osmolarity, which depend on the accumulation of tissue osmolytes, particularly intracellular lactate (Okada et al., 2016). The prolonged T2 value in the patient group may have been due to the increased acidification tendency in the masseter muscle under the experimental clenching compared with the healthy control group (Chikui et al., 2010; Shiraishi et al., 2011). Several studies utilizing ^31^P‐MRS confirmed the existence of a strong relationship between transverse relaxation properties and the metabolic state in large skeletal muscles, such as the gastrocnemius or the wrist flexor muscles, engaged in exercise (Hoff et al., 2013; Mizuno et al., 1994; Segal, 2007; Stutzig et al., 2016; Vandenborne et al., 2000). As shown in semi‐quantitative evaluation of the pH, that in the skeletal class III patients was significantly lower than that of the healthy control group at rest and recovery in the present study. This suggests a low metabolic efficiency of the phosphate compounds in the masseter muscle of the patient group during resting periods. Thus, both ^31^P‐MRS and T2‐weighted MR images would be useful for evaluating the fatigue of masseter muscles. The most interesting finding of the present study is that patients with mandibular prognathism were shown to have more fatigue in their masseter muscle than the healthy control group in the resting phase, but not during clenching, suggesting that the maxillofacial morphology and muscle fatigue may be related in daily activities.

^31^P‐MRS and T2‐weighted MR images of the resting muscles seem to be a useful method for assessing masseter fatigue in patients with maxillofacial skeletal deformity. It might also be useful as an additional tool for diagnosing and monitoring neuromuscular and metabolic diseases. These findings suggest that interindividual variations in the masseter fatigue based on the energy metabolism can be differentiated by not only the T2 relaxation value but also the signal intensity of high‐phosphate compounds. Prolongation of the T2 value would indicate acidification of the masseter muscles due to a decreased pH within the muscle cells (Figure 4) (Bruce et al., 2013; Kemp et al., 2014; Taylor et al., 1983), which leads to a transient change in the osmotic pressure, thereby causing water to migrate from outside to inside the cell.

The cross‐sectional area of the masseter muscle in the patients was significantly smaller than that in the healthy control group (*p* < 0.05). Ariji et al. reported that the cross‐sectional area of the masseter muscle in patients with mandibular prognathism was an average of 318.3 mm^2^, significantly smaller than that in normal subjects (an average of 368.3 mm^2^) (Ariji et al., 2001). Our results were similar to these previously reported findings. To optimize the spectral quality, we applied a circular 10‐cm‐diameter surface coil that conformed to the anatomy of the masseter muscle, and the head was stabilized with a custom‐made pillow. While the ROI was enlarged as much as possible within each individual masseter muscle, we need to fit the ROI into the cross‐sectional area of the masseter muscle. Previous studies mentioned that the methods for determining the ROI might have influenced the values by including different parts of the masseter muscle, depending on the muscle dimensions (Gregor et al., 2013; Lam & Hannam, 1992). However, it is considered that the extent of the ROI employed in the present study is suitable for smaller muscles, such as the masseter.

## 5 CONCLUSIONS

This study was performed to examine the validity of a diagnostic imaging method utilizing molecular imaging to evaluate acute and transient muscle fatigue of the masticatory muscles, which have a relatively small cross‐sectional area, and to establish a method for diagnosing masticatory muscle fatigue. In conclusion, we showed that mfMRI and ^31^P‐MRS are believed to be useful for assessing masseter fatigue.

## CONFLICT OF INTEREST

The authors declare no potential conflicts of interest with respect to the authorship and/or publication of this article.

## AUTHOR CONTRIBUTIONS

*Conceived and designed the experiments*: Masahiro Arakawa, Toru Kitahara, Masahiro Iikubo, Kenji Yuasa, and Ichiro Takahashi. *Performed the experiments*: Masahiro Arakawa, Toru Kitahara, and Daisuke Inadomi. *Analyzed the data*: Masahiro Arakawa, Toru Kitahara, and Daisuke Inadomi. *Finalize the paper*: Masahiro Arakawa, Toru Kitahara, and Ichiro Takahashi.

## Data Availability

The datasets during and/or analysed during the current study available from the corresponding author on reasonable request.
